# Changes in US Medicaid Enrollment During the COVID-19 Pandemic

**DOI:** 10.1001/jamanetworkopen.2021.9463

**Published:** 2021-05-05

**Authors:** Peggah Khorrami, Benjamin D. Sommers

**Affiliations:** 1 Department of Health Policy and Management, Harvard T.H. Chan School of Public Health, Boston, Massachusetts; 2 Department of Medicine, Brigham & Women’s Hospital, Boston, Massachusetts

## Abstract

This cross-sectional study analyzes changes in Medicaid enrollment for all 50 US states and Washington, DC, during the first 9 months of 2020, at the beginning of the COVID-19 pandemic.

## Introduction

The COVID-19 pandemic has led to unemployment among millions of US adults.^1^ Research shows that the Patient Protection and Affordable Care Act and Medicaid provide an important safety net for coverage after job losses,^2^ but it is unclear how changes in Medicaid enrollment during the pandemic vary across states and what policy factors are associated with those changes. The objective of this study is to analyze changes in Medicaid enrollment for all 50 US states and Washington, DC, during the first 9 months of 2020 and to test for associations with state Medicaid expansion, pandemic application simplification steps, and changes in the unemployment rate.

## Methods

This cross-sectional study was deemed as non–human participants research by the Harvard University institutional review board, which waived the need for informed consent because all data were deidentified and publicly available. This study follows the Strengthening the Reporting of Observational Studies in Epidemiology (STROBE) reporting guidelines.

We analyzed Medicaid enrollment data from the Centers for Medicare & Medicaid Services for all 50 states and Washington, DC, from January 2019 through September 2020, the most recent month with available data. We plotted descriptive monthly trends for enrollment expressed as a percentage of each state’s 2019 population obtained from the US Census Bureau, with the sample stratified into expansion and nonexpansion states^3^ and with estimates weighted by state population. Then we estimated linear regression models for the change in Medicaid enrollment from the beginning of our study period (January 2019) to the end (September 2020); sensitivity analyses assessed different time frames. We specified univariable and multivariable models examining 3 independent variables: Medicaid expansion, change in state unemployment rates during the study period from the Bureau of Labor Statistics, and the number of Medicaid enrollment simplification steps taken by each state during the pandemic, including extending presumptive eligibility to additional eligibility groups and more lenient residency criteria for individuals temporarily residing out of state because of the pandemic (see eMethods in the Supplement for full details and regression models).^4^

A linear regression was conducted with a significance threshold of *P* < .05 (2-sided). The analysis was done with Stata statistical software version 14.0 (StataCorp) in January 2021.

## Results

From January 2019 to September 2020, Medicaid enrollment increased from 48.2 million to 51.8 million individuals in expansion states and from 17.2 million to 18.8 million individuals in nonexpansion states. The Figure shows changes in the percentage of the population enrolled in Medicaid, for expansion vs nonexpansion states. Panel A of the Figure shows that enrollment was flat until it began to increase in March 2020 in both expansion and nonexpansion states. Between January 2019 and September 2020, enrollment increased by 1.4 percentage points in nonexpansion states and 1.6 percentage points in expansion states. Panel B of the Figure plots the net change in Medicaid enrollment vs the change in the state unemployment rate. The largest changes were in Idaho (which expanded starting January 2020) and Kentucky. The regression line for Medicaid growth had a modest negative slope with respect to unemployment increases, in both expansion and nonexpansion states.

**Figure.**
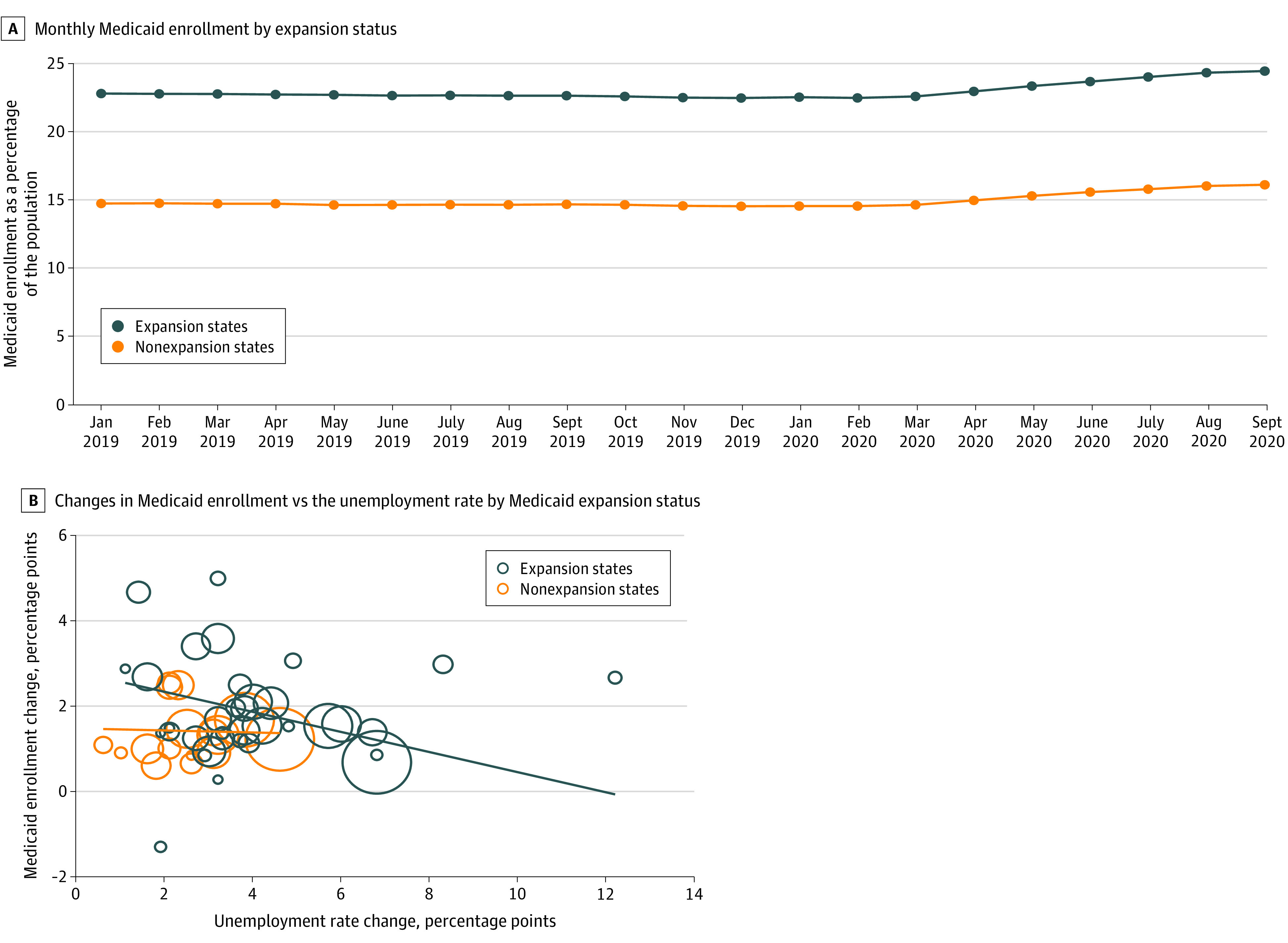
Changes in State Medicaid Enrollment, January 2019 to September 2020 A, Monthly Medicaid enrollment by expansion status. B, Changes in Medicaid enrollment vs the unemployment rate, by Medicaid expansion status. In B, the size of the circles is proportional to state population, and lines show regression model slope for unemployment vs enrollment changes, stratified by expansion state status.

The Table shows regression results. Enrollment gains were significantly negatively associated with increases in unemployment, in both unadjusted (estimate, −0.14 percentage point; 95% CI, −0.27 to −0.00 percentage point; *P* = .046) and adjusted (estimate, −0.20 percentage point; 95% CI, −0.34 to −0.06 percentage point; *P* = .007) models. Medicaid expansion was associated with higher enrollment growth in the adjusted model (estimate, 0.68 percentage point; 95% CI, 0.07 to 1.29 percentage points; *P* = .03), whereas application simplification steps were not associated with enrollment changes. Results were similar when analyzing 12-month changes from September 2019 to September 2020, or from January 2020 to September 2020.

**Table.** State-Level Factors Associated With Changes in Medicaid Enrollment, January 2019 to September 2020^a^

Variable	Univariable analysis		Multivariable analysis	
	Estimate (95% CI), percentage points	*P* value	Estimate (95% CI), percentage points	*P* value
Medicaid expansion	0.32 (−0.18 to 0.81)	.21	0.68 (0.07 to 1.29)	.03
Change in unemployment rate	−0.14 (−0.27 to −0.00)	.046	−0.20 (−0.34 to −0.06)	.007
Application simplification changes				
0	1 [Reference]	NA	1 [Reference]	NA
1	0.29 (−0.48 to 1.05)	.46	−0.01 (−0.83 to 0.80)	.97
≥2	−0.04 (−0.57 to 0.49)	.89	−0.22 (−0.80 to 0.36)	.44

Abbreviation: NA, not applicable.

^a^ Outcomes are expressed as the percentage point change in a state’s population enrolled in Medicaid between January 2019 and September 2020. Data are shown for 50 states plus Washington, DC.

## Discussion

Medicaid enrollment increased as the US’s COVID-19 pandemic and economic shutdown began in March 2020, with approximately 5 million more people covered nationally by September 2020. This increase occurred in both expansion and nonexpansion states, as found in a previous shorter-term analysis,^5^ although we found evidence suggesting that growth was larger in expansion states. Enrollment simplification steps were not associated with Medicaid growth. Unexpectedly, we found that enrollment growth was greater in states with smaller changes in unemployment in 2020. This may indicate that Medicaid growth is associated with factors other than job loss, including reduced work hours making more people eligible, greater focus on health care during the pandemic, and the maintenance of effort requirement passed by Congress in March 2020, which offered states more funding in exchange for a requirement that they not disenroll anyone from Medicaid during the public health emergency.^6^ Limitations of this study include the fact that we have data only through the fall of 2020, a lack of information on uninsured rates, and a regression model with 51 state-level observations, which precluded a detailed analysis of enrollment policies and was subject to potential unmeasured confounders.

## Notes

Supplement. **eMethods.**

## References

[1] Berkowitz SA, Basu S. Unemployment insurance, health-related social needs, health care access, and mental health during the COVID-19 pandemic. JAMA Intern Med. Published online November 30, 2020. doi:10.1001/jamainternmed.2020.7048 33252615 PMC8094006

[2] Agarwal SD, Sommers BD. Insurance coverage after job loss: the importance of the ACA during the Covid-associated recession. N Engl J Med. 2020;383(17):1603-1606. doi:10.1056/NEJMp2023312 32813967

[3] Centers for Medicare & Medicaid Services. State Medicaid and CHIP applications, eligibility determinations, and enrollment data. Updated January 15, 2021. Accessed January 14, 2021. https://data.medicaid.gov/Enrollment/State-Medicaid-and-CHIP-Applications-Eligibility-D/n5ce-jxme

[4] Kaiser Family Foundation. Medicaid emergency authority tracker: approved state actions to address COVID-19. Published 2020. Accessed September 8, 2020. https://www.kff.org/coronavirus-covid-19/issue-brief/medicaid-emergency-authority-tracker-approved-state-actions-to-address-covid-19/

[5] Frenier C, Nikpay SS, Golberstein E. COVID-19 has increased Medicaid enrollment, but short-term enrollment changes are unrelated to job losses. Health Aff (Millwood). 2020;39(10):1822-1831. doi:10.1377/hlthaff.2020.00900 32757955

[6] Allen HL, Sommers BD. Medicaid and COVID-19: at the center of both health and economic crises. JAMA. 2020;324(2):135-136. doi:10.1001/jama.2020.10553 32525506

