# Supplementary material for: Changes in US Medicaid Enrollment During the COVID-19 Pandemic
Source: JAMA Netw Open. 2021 May 5;4(5):e219463. doi: 10.1001/jamanetworkopen.2021.9463 (PMC8100862; doi:10.1001/jamanetworkopen.2021.9463)
Supplement: Supplement. — eMethods. [file jamanetwopen-e219463-s001.pdf]

Supplemental Online Content

Khorrami P, Sommers BD. Changes in US Medicaid enrollment during the COVID-19 pandemic. *JAMA Netw Open*. 2021;4(5):e219463.
doi:10.1001/jamanetworkopen.2021.9463

eMethods.

This supplemental material has been provided by the authors to give readers additional information about their work.

© 2021 Khorrami P et al. *JAMA Network Open.*

eMethods

Regression Model

Our regression analysis used univariate and multivariate linear regression models. The multivariate model equation was as follows:

$$MedicaidPctGrowth = \beta_0 + \beta_1 Expansion + \beta_2 UnemploymentPctChange + \beta_3 PolicyIndexOne + \beta_4 PolicyIndexTwo$$

where *MedicaidPctGrowth* is the percent Medicaid enrollment change as a share of the state population between January 2019 and September 2020, *Expansion* is an indicator for Medicaid expansion in a state, *UnemploymentPctChange* is the percent change in the state unemployment rate between January 2019 and September 2020, *PolicyIndexOne* is an indicator for a state taking one Medicaid enrollment simplification step during the pandemic, and *PolicyIndexTwo* is an indicator for a state taking two or more enrollment simplification steps.

The following criteria were used as measures of state-related simplification of Medicaid enrollment procedures:

1) Accept self-attestation for all eligibility criteria except citizenship and immigration status

2) Apply less restrictive or otherwise modify income, resource and other financial eligibility requirements

3) Expand the definition of temporary absence to establish residency for individuals temporarily out of state due to the emergency

4) Extend reasonable opportunity period to verify immigration status

5) Extend hospital presumptive eligibility to non-MAGI eligibility groups

6) Extend presumptive eligibility to additional eligibility groups

7) Increase number of presumptive eligibility periods in a 12-month period

The distribution of state-related simplification procedures was as follows:

Number of Simplification Procedures	Expansion	Non-Expansion	Total
0	9	9	18
1	1	9	10
2	3	8	11
3	2	5	7
4	1	3	4
5	0	1	1
6	0	0	0
7	0	0	0
**Total**	16	35	51

© 2021 Khorrami P et al. *JAMA Network Open.*
